# Low omega-3 index values and monounsaturated fatty acid levels in early pregnancy: an analysis of maternal erythrocytes fatty acids

**DOI:** 10.1186/s12944-018-0716-6

**Published:** 2018-04-02

**Authors:** Axelle Hoge, Florence Bernardy, Anne-Françoise Donneau, Nadia Dardenne, Sylvie Degée, Marie Timmermans, Michelle Nisolle, Michèle Guillaume, Vincenzo Castronovo

**Affiliations:** 1 Department of Public Health, University of Liège, Avenue Hippocrate 13 - B23, 4000 Liège, Belgium; 2 Department of Obstetrics and Gynecology, CHR Citadelle Hospital, University of Liège, Liège, Belgium; 3 Metastasis Research Laboratory, GIGA-CANCER, University of Liège, Liège, Belgium

**Keywords:** Erythrocyte, Fatty acid, Pregnancy

## Abstract

**Background:**

It is unanimously recognized that the maternal nutritional status at the pregnancy onset influence both short-term and long-term health of the mother and offspring. Among several nutrients, LCPUFA, particularly from the omega-3 family, are of utmost importance. This study was carried out to determine fatty acids profile of maternal erythrocyte membranes in early pregnancy and to identify potential determinants impacting on this status.

**Methods:**

A cohort of 122 healthy women with a singleton pregnancy was included. Fatty acids were analyzed using gas chromatography. Because of the lack of cutoff values, reference ranges were used to determine fatty acids categories.

**Results:**

Of concern, our data revealed low monounsaturated and long-chain omega-3 fatty acid status in most participants. More than 75% of Belgian pregnant women exhibited Pal, AO and EPA levels as well as IOM3 values below the laboratory reference ranges. Higher DHA concentrations and IOM3 values were found among foreign-nationality participants, non-smokers and physically active women. With regard to dietary factors, omega-3 supplements and diet seem to be complementary since DHA from supplements (but not from diet) and EPA from diet (but not from supplements) were found to be associated with higher concentrations of DHA and EPA, respectively.

**Conclusions:**

Our study presents evidence demonstrating that the fatty acid status of most early pregnant women is far from being optimal based on the admitted general reference values. Clinicians should be advice to carefully evaluate and improve this status to guarantee the best possible outcome for both the mother and the baby.

**Electronic supplementary material:**

The online version of this article (10.1186/s12944-018-0716-6) contains supplementary material, which is available to authorized users.

## Background

Nutrition before and during pregnancy has a crucial impact on maternal outcomes and fetal growth and development. Considerable evidence has accumulated to show that impaired nutritional environment in early life exerts long-term health effects, a phenomenon known as nutritional programming of disease [1, 2].

Fatty acids, especially their more unsaturated derivatives, affect many physiological functions, involving a wide spectrum of mechanisms of action [3]. Beyond sources of metabolic and storage energy, polyunsaturated fatty acids (PUFA) confer unique structural and functional properties to cell membranes, regulate inter- and intracellular communication as well as gene expression [4, 5]. Several specific fatty acids regulate transcription through the peroxisome proliferator-activated receptors family (PPARs). Of particular interest, studies revealed a possible role of PPARs in placental metabolism, fetal development and pre-eclampsia [6, 7].

The brain is the second fattiest organ of the body with over 60% of its structural material, a highly specialized fat rich in phospholipids and cholesterol [8]. Most of its development occurs during fetal life. While birth weight is only 5% of an adult, the newborn brain is about 70% of an adult size [9]. Long-chain polyunsaturated fatty acids (LCPUFA) are selectively transferred from the mother to the fetus and are accumulated in the fetal nervous system as early as the second part of pregnancy. In particular, docosahexaenoic acid (DHA) is the predominant fatty acid in the brain tissue, particularly in the synaptic membranes and retina [8, 10]. A large number of studies have shown that sufficient LCPUFA availability during fetal life can positively affect visual and neurodevelopmental outcomes [11–14].

In addition to the best known roles in neural development of infants, several studies suggest implications of PUFA in a number of pregnancy conditions and birth outcomes: reduction of preterm birth and low birth weight [15, 16], decreased risk of pre-eclampsia [15, 17, 18] and perinatal depression [19], as well as the primary prevention of asthma and allergies in early childhood and childhood [20].

During gestation, fatty acids accumulated in developing tissues depend on placental transfer which in turn is influenced by maternal diet and metabolism [21]. Maternal PUFA supply is critical to determining PUFA status of both the mother and the newborn [22, 23].

The Western pattern diet however suggests that pregnant women may have difficulties to meet the fetal PUFA requirements. Several studies examined the fatty acid status of mothers by analyzing plasma phospholipid fatty acid content. Focusing mainly on the PUFA and their longer chain derivatives, they observed a continuous decline of their relative amounts throughout pregnancy suggesting that mothers may be unable to meet the fetal demands for these fatty acids [24, 25]. Subsequent depletion in maternal DHA stores has been associated with an increased risk of postpartum depression [26], which can negatively affect mother-child interaction. Normalization of postpartum levels is slow. Maternal DHA status seems to turn to repletion over a period of 6 months to 1 year after delivery [27]. Another specific focus of scientific interest is the *trans* fatty acid (tFA) intake during pregnancy since these tFA could alter the transplacental passage of LCPUFA by using the same binding sites in placental membranes and affect the metabolism of PUFA [28, 29]. *Trans* fatty acids could prevent the conversion of linoleic acid (LA) and alpha-linolenic acid (ALA) to arachidonic acid (AA) and DHA, respectively [30]. A negative association has been observed between tFA and plasma LCPUFA in both the mother at different times of pregnancy [31] and the newborn [32, 33].

While PUFA composition of plasma phospholipids reflects short-term intake of PUFA and is subject to high biological variability, erythrocyte fatty acid composition appears to be more appropriate to represent an integrative measure of the fatty acid status over the previous few months [34–37]. Moreover, it has been suggested that maternal erythrocyte phospholipids are involved in the placental transfer of DHA and AA to the fetus [38].

The assessment of the nutritional status of fatty acids in the first trimester of pregnancy is highly relevant considering that the onset of neurogenesis occurs early after conception. Vlaardingerbroek & Hornstra concluded that neonatal essential fatty acid and LCPUFA status might be predicted on the basis of essential fatty acid and LCPUFA concentrations of maternal erythrocyte phospholipids measured early in pregnancy [39]. All together, these data suggest that the fatty acid status at the early stages of pregnancy may have a significant impact on pregnancy outcomes. To our knowledge, no study has undertaken a comprehensive evaluation of the maternal fatty acid status in early pregnancy or an exploration of potential determinants that could influence this status. This study was designed to address these questions and draw clinician’s attention to the importance of these key nutritional parameters.

## Methods

### Study design and population

This study which took place between February and August 2016 was initiated from a prospective cohort study aimed to investigate the relationship between maternal erythrocyte fatty acids and pregnancy and birth outcomes. The study protocol was reviewed and approved by the Ethics Committee of the CHR Citadelle Hospital of Liège, Belgium (B412201526650).

Women were recruited from the Obstetrics and Gynecology Department of the CHR Citadelle Hospital, Liège, Belgium at their first antenatal appointment. Inclusion criteria were (1) being between the 7th and 18th week of gestation; (2) being free from any chronic diseases such as hypertension and diabetes; (3) presenting with singleton pregnancy. From all eligible subjects, 122 women agreed to participate and signed the informed consent.

Sociodemographic, anthropometric and lifestyle characteristics of participants, including age, nationality, level education, smoking status and physical activity, were collected by a self-administered questionnaire. Briefly, level of education was expressed as low (primary and lower and upper secondary) and high (non-university degree and university degree). Body mass index (BMI) was calculated as weight (kg) divided by height squared (m^2^). Gestational age and parity were obtained from the medical record. Detailed information on use of omega-3 fatty acid supplements was also collected, including product name, type of medication, dose, frequency and duration of use. Daily intake from supplement for EPA and DHA was calculated by multiplying the daily frequency of use by the nutrient composition per dose. A food survey was also conducted on a subsample of 29 pregnant women using a previously validated food frequency questionnaire (FFQ) reporting the consumption frequency and portion size of 167 food items over the last 3 months [40, 41]. FFQ-estimated daily intake of each fatty acid was calculated by multiplying the consumption frequency of each food item by the specific fatty acid content of selected portions. Fatty acid contents were obtained from the French ‘SUpplementation en VItamines et Mineraux AntioXydants’ (SU.VI.MAX) Food Composition Database [42]. Finally, fasting blood samples were taken on each participant in EDTA-containing tubes, refrigerated and brought in an ice batch to a medical analysis laboratory within 24 h.

### Sample preparation and erythrocyte fatty acid analysis

Plasma and erythrocyte were separated by centrifugation at 3600 rpm for 5 min. The erythrocyte fraction was washed twice with a normal saline solution and was stored at − 80 °C until further analysis.

Erythrocyte fatty acids were analyzed by gas-chromatography after their derivatization to methyl esters (FAME), due to their higher volatility. Fatty acid methyl esters were obtained in an acid catalyzed reaction, using methanol as derivatization agent and hydrochloric acid as catalyst. An exact volume of 40 μl washed erythrocytes was diluted into 0.4 ml hexane and 0.4 ml methanolic HCl. The solution was placed in an oven at 95 °C for 2 h. After solution was cooled down at room temperature, FAME were extracted into 3 ml n-hexane, 0.8 ml saturated NaHCO_3_ solution and 1 ml double-distilled water. The organic phase was completely evaporated to dryness under vacuum and the residue was dissolved into 0.5 ml n-hexane. The resulting solution was poured into a brown glass flask before injection through gas chromatograph. FAME were quantified using an Agilent 7890A gas chromatograph (Agilent Technologies Inc., USA).

Fifteen fatty acids were measured and expressed as percentages of total fatty acids. The laboratory reference values (RV) established on healthy adults were 0.16–0.40% for myristic acid, 21.2–23.5% for palmitic acid, 13.4–17.2% for stearic acid, 0.18–0.49% for palmitoleic acid, 0.67–0.96% for *cis* vaccenic acid, 12.0–14.7% for oleic acid, 8.49–11.2% for linoleic acid, 0.04–0.09% for gamma-linolenic acid, 1.28–2.20% for dihomo-gamma-linolenic acid, 11.0–13.4% for arachidonic acid, 21.9–25.4% for total n-6 PUFA, 0.08–0.17% for alpha-linolenic acid, 0.75–2.34% for eicosapentaenoic acid, 5.27–8.87% for docosahexaenoic acid, ≤ 0.17% for *trans* vaccenic acid and ≤ 0.07% for elaidic acid. The omega-3 index as well as n-6/n-3, AA/EPA and LA/DGLA ratios were determined to describe the functional PUFA status. The omega-3 index was defined as erythrocyte EPA plus DHA expressed as weight percentage of total fatty acids [43]. RV were 7.50–10.0% for omega-3 index, 1.00–4.00 for n-6/n-3, 5.00–10.0 for AA/EPA and ≤ 4.40 for LA/DGLA.

### Statistical analysis

Results were expressed as mean ± standard deviation (SD) or as median and interquartile range (IQR) for skewed data. For categorical variables, frequency tables were used. Pearson correlation, or the Spearman’s rank correlation, was used to assess the associations between two quantitative variables. Means between groups were compared by the Student’s t-test and the one-way analysis of variance (ANOVA). Chi-square test was applied to compare proportions of categorical variables between groups. Results were considered significant at the 5% critical level (*P* < 0.05). All statistical analyses were performed with SAS 9.4 (© SAS Institute Inc., Cary, NC, USA).

## Results

### Subjects

The characteristics of the study subjects are described in Table 1. Women were 28.3 ± 5.6 years old and 62.3% had a low educational level. There were 68 (55.7%) Belgians, 19 (15.6%) came from North Africa or Middle East, 9 (7.4%) from Central Africa, 8 (6.6%) from Asia, 7 (5.7%) from West Africa, 7 (5.7%) from South Europe, 3 (2.5%) from West Europe and 1 (0.8%) from East Africa. Of the 108 non-smokers, 19 pregnant women reported smoking in the past. Nine participants stopped smoking due to their pregnancy. A quarter (24.6%) reported omega-3 fatty acid supplementation with a mean EPA intake of 38.5 ± 88.7 mg per day as well as a mean DHA intake of 184.7 ± 29.1 mg per day. The 29 (23.8%) women who completed the FFQ were comparable to the others, except for physical activity which was higher (44.8% vs. 23.7%, *p* = 0.028). Median n-6 and n-3 PUFA intake were, respectively, 15.3 (11.2–22.0) and 1.72 (1.38–2.01) g/day, yielding a dietary n-6/n-3 ratio of 8.78 (6.94–10.7). Median daily intake of EPA and DHA were 120.0 (50.0–230.0) and 150.0 (60.0–300.0) mg, respectively. Twelve participants (41.4%) reported eating at least 200 g of fish per week. Median intake of oily fish was 63.0 (7.00–154.0) g/week.

**Table 1:** Maternal characteristics (*n* = 122)

Variable	Summary statistics^a^
Age (years)	28.3 ± 5.6
Gestational age (weeks)	10.9 ± 2.6
Parity	
Nulliparous	51 (41.8)
Primiparous	41 (33.6)
Multiparous	28 (23.0)
Not know	2 (1.6)
BMI class (kg/m^2^)	
< 25	76 (62.3)
25–30	33 (27.1)
≥ 30	13 (10.7)
Nationality	
Belgian	68 (55.7)
Other	54 (44.3)
Level of education	
Low	76 (62.3)
High	46 (37.7)
Smoking status	
Non-smoker	108 (88.5)
Smoker	14 (11.5)
Physical activity	
Yes	35 (28.7)
No	87 (71.3)
n-3 PUFA supplement use	
Yes	30 (24.6)
No	92 (75.4)
EPA supplement intake (mg/day)	38.5 ± 88.7
DHA supplement intake (mg/day)	184.7 ± 29.1

^a^Data are presented as mean ± SD or number (%)

EPA eicosapentaenoic acid, DHA docosahexaenoic acid

### Maternal erythrocyte fatty acid status

The fatty acid status in maternal erythrocyte phospholipids at early pregnancy is shown in Table 2. The predominant saturated fatty acid was palmitic acid, with a mean concentration of 21.8 ± 1.02%. Oleic acid was the most abundant monounsaturated fatty acid: 11.0 ± 0.94%. Total n-6 PUFA was four times the total n-3 PUFA, AA and LA being the predominant PUFA.

**Table 2:** Maternal FA composition (% of total FA) and FA category defined by the reference values (RV)

Fatty acids (%)		Mean ± SD	Below RV	Within RV	Above RV
SFA					
Myristic acid	C14:0	0.29 ± 0.08	1 (0.8)	110 (90.2)	11 (9.0)
Palmitic acid	C16:0	21.8 ± 1.02	27 (22.1)	89 (73.0)	6 (4.9)
Stearic acid	C18:0	16.1 ± 0.76	0 (0.0)	114 (93.4)	8 (6.6)
MUFA					
Palmitoleic acid	C16:1n-7	0.15 ± 0.06	92 (75.4)	30 (24.6)	0 (0.0)
*cis* vaccenic acid	C18:1n-7	0.85 ± 0.11	1 (0.8)	107 (87.7)	14 (11.5)
Oleic acid	C18:1n-9	11.0 ± 0.94	109 (89.3)	13 (10.7)	0 (0.0)
n-6 PUFA					
Linoleic acid	C18:2n-6	8.43 ± 1.13	63 (51.6)	56 (45.9)	3 (2.5)
Gamma-linolenic acid	C18:3n-6	0.05 ± 0.01	16 (13.1)	106 (86.9)	0 (0.0)
Dihomo-gamma-linolenic acid	C20:3n-6	1.52 ± 0.35	30 (24.6)	85 (69.7)	7 (5.7)
Arachidonic acid	C20:4n-6	14.3 ± 1.26	1 (0.8)	26 (21.3)	95 (77.9)
Total n-6 PUFA		24.3 ± 1.51	8 (6.6)	89 (73.0)	25 (20.5)
n-3 PUFA					
Alpha-linolenic acid	C18:3n-3	0.13 ± 0.05	10 (8.2)	90 (73.8)	22 (18.0)
Eicosapentaenoic acid	C20:5n-3	0.51 ± 0.25	106 (86.9)	16 (13.1)	0 (0.0)
Docosahexaenoic acid	C22:6n-3	5.27 ± 1.42	67 (54.9)	53 (43.4)	2 (1.6)
Total n-3 PUFA		5.92 ± 1.59	87 (71.3)	35 (28.7)	0 (0.0)
tFA					
*trans* vaccenic acid	C18:1n-7 t	0.13 ± 0.03	NA	115 (94.3)	7 (5.7)
Elaidic acid	C18:1n-9 t	0.04 ± 0.01	NA	119 (97.5)	3 (2.5)
Omega-3 index		5.79 ± 1.60	108 (88.5)	12 (9.8)	2 (1.6)
n-6/n-3 ratio^a^		4.44 ± 1.36	0 (0.0)	53 (43.4)	69 (56.6)
AA/EPA ratio		34.5 ± 16.8	0 (0.0)	3 (2.5)	119 (97.5)
LA/DGLA ratio		5.82 ± 1.47	NA	17 (13.9)	105 (86.1)

Fatty acid composition of maternal erythrocyte phospholipids is presented as mean ± SD and maternal erythrocyte fatty acid category as number (%)

*SFA* saturated fatty acids, *MUFA* monounsaturated fatty acids, *n-6 PUFA* omega-6 polyunsaturated fatty acids, *n-3 PUFA* omega-3 polyunsaturated fatty acids, *tFA trans* fatty acids

^a^n-6/n-3 ratio = total n-6 PUFA/total n-3 PUFA

For more than 75% of women, levels of palmitoleic acid, oleic acid and eicosapentaenoic acid fell below the RV. By contrast, erythrocyte arachidonic acid was above RV in 95 subjects (77.9%). The omega-3 index (RV: 7.5–10%) was out of range for 90.1% of the participants. Respectively 119 (97.5%) and 105 (86.1%) pregnant women had high AA/EPA and LA/DGLA ratios compared to the laboratory reference values.

Pearson correlation coefficients between polyunsaturated fatty acids as well as between *trans* fatty acids and polyunsaturated fatty acids were included in Additional file 1: Table S1. Significant and relevant correlations were observed between EPA and DHA (*r* = 0.70), EPA and omega-3 index (*r* = 0.77), as well as between DHA and omega-3 index (*r* = 0.99).

### Potential determinants of maternal erythrocyte polyunsaturated fatty acid concentrations

Associations between maternal characteristics and erythrocyte polyunsaturated fatty acid concentrations are described in Table 3**.** Similarly, relations between the omega-3 index and the three studied ratios are given in Table 4.

**Table 3:** Potential determinants of maternal erythrocyte polyunsaturated fatty acid composition (% of total FA)

Variable	n-6 PUFA				n-3 PUFA		
	LA	GLA	DGLA	AA	ALA	EPA	DHA
Age (years)	***r*** **= −0.20**	*r* = 0.05	***r*** **= 0.21**	r = − 0.03	*r* = 0.14	***r*** **= 0.20**	***r*** **= 0.18**
Gestational age (weeks)	*r* = − 0.17	*r* = − 0.06	*r* = − 0.10	*r* = − 0.18	*r* = − 0.05	*r* = 0.03	***r*** **= 0.28**
Parity							
Nulliparous	8.50 ± 1.16	0.05 ± 0.01	1.48 ± 0.29	14.3 ± 1.11	0.12 ± 0.04	0.45 ± 0.18	5.07 ± 0.98
Primiparous	8.44 ± 1.18	0.05 ± 0.01	1.48 ± 0.36	14.5 ± 1.12	0.14 ± 0.06	0.56 ± 0.29	5.32 ± 1.44
Multiparous	8.42 ± 1.08	0.05 ± 0.01	1.60 ± 0.39	14.2 ± 1.57	0.13 ± 0.05	0.51 ± 0.27	5.34 ± 1.78
BMI class (kg/m^2^)							
< 25	8.39 ± 1.11	0.05 ± 0.01	1.53 ± 0.36	14.4 ± 1.26	0.13 ± 0.05	0.51 ± 0.24	5.10 ± 1.28
25–30	8.59 ± 1.28	0.05 ± 0.01	1.49 ± 0.32	13.9 ± 1.18	0.13 ± 0.05	0.56 ± 0.30	5.57 ± 1.73
≥ 30	8.28 ± 0.89	0.05 ± 0.01	1.49 ± 0.40	14.7 ± 1.34	0.12 ± 0.03	0.45 ± 0.16	5.52 ± 1.19
Nationality							
Belgian	8.28 ± 1.05	0.05 ± 0.01	**1.58 ± 0.37**	14.4 ± 1.04	**0.14 ± 0.05**	0.52 ± 0.21	**4.99 ± 1.35**
Other	8.63 ± 1.21	0.05 ± 0.01	**1.44 ± 0.31**	14.2 ± 1.50	**0.11 ± 0.04**	0.51 ± 0.29	**5.62 ± 1.43**
Level of education							
Low	8.53 ± 1.07	**0.05 ± 0.01**	1.51 ± 0.36	14.4 ± 1.36	0.13 ± 0.06	0.51 ± 0.29	5.19 ± 1.60
High	8.28 ± 1.22	**0.05 ± 0.01**	1.53 ± 0.33	14.2 ± 1.08	0.13 ± 0.04	0.53 ± 0.18	5.41 ± 1.04
Smoking status							
Non-smoker	8.49 ± 1.15	0.05 ± 0.01	1.52 ± 0.36	14.2 ± 1.29	0.13 ± 0.05	0.52 ± 0.26	**5.41 ± 1.41**
Smoker	8.00 ± 0.84	0.05 ± 0.01	1.52 ± 0.23	14.8 ± 0.95	0.15 ± 0.06	0.51 ± 0.18	**4.18 ± 0.95**
Physical activity							
Yes	8.39 ± 1.28	**0.05 ± 0.01**	1.50 ± 0.33	14.1 ± 1.14	0.13 ± 0.04	0.58 ± 0.25	**5.81 ± 1.49**
No	8.45 ± 1.07	**0.05 ± 0.01**	1.52 ± 0.36	14.4 ± 1.31	0.13 ± 0.05	0.49 ± 0.25	**5.05 ± 1.33**
n-3 PUFA supplement use							
Yes	8.34 ± 1.19	0.05 ± 0.01	1.50 ± 0.32	14.3 ± 1.71	0.13 ± 0.04	0.55 ± 0.22	5.52 ± 1.37
No	8.46 ± 1.12	0.05 ± 0.01	1.52 ± 0.36	14.3 ± 1.22	0.13 ± 0.05	0.50 ± 0.26	5.19 ± 1.43
EPA supplement intake (mg/day)	*r* = − 0.20	*r* = − 0.11	*r* = − 0.03	*r* = 0.12	r = − 0.05	r = 0.28	*r* = − 0.01
DHA supplement intake (mg/day)	***r*** **= − 0.41**	*r* = − 0.04	*r* = 0.04	*r* = − 0.26	r = − 0.27	***r*** **= 0.56**	***r*** **= 0.57**

Data are presented as mean ± SD or as Pearson correlation coefficient

Bold font indicates *P*-values less than 0.05

**Table 4:** Potential determinants of maternal erythrocyte omega-3 index and n-6/n-3, AA/EPA and LA/DGLA ratios

Variable	Index	Ratio		
	IOM3	n-6/n-3	AA/EPA	LA/DGLA
Age (years)	***r*** **= 0.19**	*r* = −0.16	***r*** **= − 0.19**	***r*** **= − 0.29**
Gestational age (weeks)	***r*** **= 0.25**	***r*** **= − 0.27**	*r* = 0.06	r = − 0.01
Parity				
Nulliparous	5.52 ± 1.08	4.50 ± 1.08	37.9 ± 17.7	5.95 ± 1.46
Primiparous	5.89 ± 1.67	4.40 ± 1.41	32.4 ± 16.6	5.97 ± 1.56
Multiparous	5.86 ± 2.00	4.54 ± 1.61	34.8 ± 16.8	5.53 ± 1.38
BMI class (kg/m^2^)				
< 25	5.61 ± 1.45	4.57 ± 1.41	35.3 ± 17.9	5.74 ± 1.47
25–30	6.13 ± 1.99	4.22 ± 1.36	31.7 ± 15.5	5.98 ± 1.47
≥ 30	5.96 ± 1.25	4.21 ± 1.01	36.8 ± 13.1	5.91 ± 1.57
Nationality				
Belgian	**5.52 ± 1.50**	4.59 ± 1.30	**31.6 ± 12.1**	**5.48 ± 1.32**
Other	**6.12 ± 1.67**	4.25 ± 1.43	**38.1 ± 20.9**	**6.26 ± 1.55**
Level of education				
Low	5.70 ± 1.83	**4.63 ± 1.54**	**37.2 ± 19.5**	5.95 ± 1.47
High	5.93 ± 1.14	**4.13 ± 0.93**	**29.9 ± 9.81**	5.61 ± 1.46
Smoking status				
Non-smoker	**5.93 ± 1.61**	**4.33 ± 1.36**	34.6 ± 17.1	5.87 ± 1.49
Smoker	**4.68 ± 1.04**	**5.26 ± 1.15**	33.7 ± 14.7	5.42 ± 1.28
Physical activity				
Yes	**6.39 ± 1.68**	**3.95 ± 1.15**	**28.9 ± 12.8**	5.89 ± 1.68
No	**5.54 ± 1.51**	**4.64 ± 1.40**	**36.7 ± 17.8**	5.80 ± 1.39
n-3 PUFA supplement use				
Yes	6.07 ± 1.51	4.21 ± 1.47	31.4 ± 17.8	5.76 ± 1.33
No	5.69 ± 1.63	4.52 ± 1.33	35.5 ± 16.5	5.84 ± 1.52
EPA supplement intake (mg/day)	r = 0.03	*r* = − 0.01	*r* = − 0.08	*r* = − 0.14
DHA supplement intake (mg/day)	***r*** **= 0.60**	***r*** **= − 0.70**	***r*** **= − 0.67**	*r* = − 0.33

Data are presented as mean ± SD or as Pearson correlation coefficient

Bold font indicates *P*-values less than 0.05

Erythrocyte DHA and omega-3 index were influenced by many factors. Higher concentrations of DHA were found among foreign-nationality participants, non-smokers and physically active women. The same trends were observed for the omega-3 index. There was no statistically significant difference between fatty acid levels of n-3 PUFA supplement users and non-users. But among supplement users, DHA supplement intake was positively correlated with EPA and DHA levels and omega-3 index, and negatively correlated with LA levels and n-6/n-3 and AA/EPA ratios.

Association between dietary intake of fatty acids and their erythrocyte phospholipid composition was also investigated. Significant correlations were observed between erythrocyte EPA concentration and EPA intake (r_s_ = 0.47; *p* = 0.0101) as well as oily fish consumption (r_s_ = 0.49; *p* = 0.0064).

## Discussion

The current study reports comprehensive fatty acid composition of maternal erythrocyte in early pregnancy. Fetal growth and development in this period are the most active and erythrocyte membrane phospholipids is a more reliable biomarker medium of fatty acid intake [44]. To the best of our knowledge, this is the first assessment of fatty acid status over a period of three months in an urban community of pregnant women in Belgium. It should be noted though that there were slightly more foreign-nationality women than in Walloon population (44% vs. 11%) [45] and the number of subjects with a low educational level was also higher (62% vs. 54%) than in the NESCaV survey [46].

Since no established cutoff values exist for fatty acid status in pregnant women, erythrocyte fatty acid concentrations were compared to the reference values provided by the laboratory and to those of the few studies which reported maternal erythrocyte fatty acid levels early in the pregnancy [39, 47–49]. Overall, Belgian pregnant women exhibited similar mean fatty acid concentrations, except for myristic acid and the three measured MUFA. *Trans* vaccenic and elaidic acids composition was not determined in these four studies. Levels of myristic and MUFA were found to be lower than in the Scottish, Dutch and British women. When reference ranges of SFA and MUFA are used, it is interesting to note that a majority of pregnant women did not reach the range values defined for palmitoleic and oleic acids (75% and 89%, respectively). SFA and MUFA have attracted much less attention as compared to PUFA in the extant literature. Mennitti et al. have recently reviewed that SFA and MUFA maternal consumption seems to be related with adverse (SFA) and beneficial (MUFA) metabolic consequences in the offspring [50]. Two recent studies investigated the relationships between erythrocyte MUFA during first trimester of pregnancy and birth outcomes. While one have observed that oleic acid and MUFA predicted low birthweight [49], the other found no association between MUFA and birth outcome measures (birth weight, birth length, head circumference and chest circumference at birth) [51]. It is worth noting that mean MUFA levels of the second study were similar to those of our study, in contrast to the first which were higher. Some studies have explored the impact of Mediterranean diet, which is low in saturated and omega-6 fatty acids but high in plant monounsaturated fat, during pregnancy on health outcomes. Even if more evidence is still required, the adherence to this eating pattern has been associated with a lower incidence of gestational diabetes [52], better fetal size parameters [53, 54], a decreased risk of asthma and atopy in childhood [55] as well as a decreased risk of spina bifida in the offspring [56]. Because of their involvement in health in diverse populations [57, 58], the role of SFA and MUFA on both short-term and long-term health of the mother and offspring should be further investigated.

With regard to PUFA concentrations, another study reported maternal erythrocyte fatty acid composition in first trimester of pregnancy in Iceland, where community is characterized by a traditional fish and cod liver oil consumption [59]. Not surprisingly, the mean n-6/n-3 ratio observed in our sample was much higher (more than 1.5 times) than in Icelandic pregnant women. The two families of PUFA compete for metabolism by desaturation enzymes and because they may influence the kind of eicosanoids that will be formed, a balance between them is a critical factor for health throughout the life cycle [60]. Prostaglandins play a central role in many processes of initiation and regulation of parturition including vasodilatation, placental blood flow, cervical ripening, and onset of labor [15, 61]. Relaxation of the uterine muscle and improvement of placental blood flow induced by the preferential formation of anti-inflammatory and vasodilating eicosanoids would delay the onset of labor [1]. From the four studies conducted in early pregnancy, only one study looked at omega-3 index (erythrocyte EPA + DHA), which was similar to that observed in our study. Consistent with what was obtained for EPA, a majority (89%) of pregnant women in our study did not reach 7.5% reference level for omega-3 index. In cardiology, the omega-3 index was proposed as a marker, and even as a risk factor, for cardiovascular events (e.g. sudden cardiac death) and a level ≥ 8% was suggested as optimal [62, 63]. When this threshold was applied to our results, 112 women (91.8%) exhibited a low long-chain omega-3 fatty acid status. With regard to pregnancy and birth outcomes, omega-3 index has been associated with the risk of post-partum depression [64] and low birthweight and preterm delivery [49, 65]. Additional trials are required to clarify if this threshold of ≥8% could also ensure optimal maternal health as well as fetal growth and development.

To the best of our knowledge, no study has reported erythrocyte AA/EPA and LA/DGLA ratios in pregnant women. The ratio between EPA and AA was found to be positively correlated with the ratio of inflammatory eicosanoids [66], which serve important regulatory functions as described above. An imbalance between EPA and AA was found to be associated with health outcome as described previously [66–68]. The ratio between LA and DGLA, was used because a deficit of delta-6-desaturase activity is accompanied by a decrease in the conversion of LA to DGLA, resulting in higher LA/DGLA values [69, 70]. Delta-6 desaturase is widely regarded as the rate-limiting enzymatic step of this fatty acid metabolic pathway. A deficit in its activity indicates that the pregnant women are no longer able to convert effectively LA and ALA, resulting in a deficiency of their longer-chain derivatives. Conversion enzymes seems to be present in the fetus, but their activity appears to be low [71]. Worryingly, in our study population, only 3% and 14% women showed AA/EPA and LA/DGLA ratios in the reference range, respectively. Further studies are required to extend the application of these ratios to the area of pregnancy health.

Maternal erythrocyte *trans* vaccenic and elaidic acids were measured in other studies but at delivery time [32, 72, 73]. Compared with their results, mean levels observed in our study population were similar for *trans* vaccenic acid but much lower (3 and 7 times lower according to the study) for elaidic acid. Currently, the effects of excessive consumption of tFA during pregnancy have been studied in great part in animals. It appears to be associated with adverse effects related to fetal and infant growth and development as well as complications in pregnancy, probably due in part to an alteration in the bioavailability of LCPUFA [50, 74] and/or a misbalance in PPARs activation. In humans, Cohen et al. observed that an excessive intake of 16:1(n-7 t) and 18:2(n-6tc) during the second trimester of pregnancy was associated with greater fetal growth, estimated by the birth-weight-for-gestational-age. The authors suggested that the risk of overweight and diabetes could thus be increased in adulthood [75]. Maternal tFA was also associated with an increased risk of fetal loss and preeclampsia [73, 76].

### Correlation between maternal erythrocyte fatty acid concentrations

As expected, there was a significant positive correlation between EPA and DHA, and between these 2 fatty acids and omega-3 index. As reported elsewhere [64], the association was stronger between DHA and omega-3 index than between EPA and omega-3 index. Contrary to previous studies [30, 31], we did not observe any inverse association between *trans* fatty acids and both n-6 (e.g. AA) and n-3 PUFA (e.g. DHA). However, since different lipid fractions were used (e.g., plasma FA versus red blood cell membrane FA) and different individual *trans* fatty acids were investigated, comparison is difficult.

### Potential determinants of maternal erythrocyte polyunsaturated fatty acid status

Several maternal characteristics, including gestational age, socioeconomic status, education, smoking and supplement use, have already been shown to affect fatty acid levels [63, 77]. In our study, nationality, smoking status, physical activity and DHA supplement intake were found to affect several erythrocyte fatty acid species. One unexpected finding of this study was that higher DHA concentrations, and consequently higher omega-3 index, were found among pregnant women of foreign-nationality. Among all studied determinants, only smoking practices were not the same between Belgian and foreign-nationality participants and could explain the differences observed in status. In fact, the percentage of Belgian women was significantly higher in the smoker group (17.7% vs. 3.7%, *p* = 0.16). Our results are in line with previous studies that have suggested that a DHA supplement intake of 200 mg/day is a suitable dose to improve erythrocyte DHA concentrations of pregnant women with a low habitual consumption of fish [78]. Despite the limited number of women who have completed the FFQ, it is worth noting that dietary EPA intake as well as oily fish consumption were found to have an impact on erythrocyte EPA concentration. These results support guidelines which emphasize the importance of consuming fish during pregnancy since dietary intake of EPA would compensate the low EPA intake from supplement observed in our study. Further research is required to increase understanding of the factors that are associated with inadequate erythrocyte fatty acid status. Identification of risk group could help to inform and target dietary recommendations for a population with specific physiological needs.

Among the strengths of this study, we highlighted the fact that the recruitment did not induce an overrepresentation of subjects with a high educational level in contrast to what is usually observed in most studies. All data were collected by the same trained researcher. All erythrocyte fatty acid analyses were performed in the same medical laboratory. However, a number of limitations should be also pointed out. Although this study reported, by far, the most comprehensive data on the FA status in pregnant women in Belgium, our sample was not perfectly representative. A larger sample size would have allowed for logistic regression model associating inadequate fatty acid status (defined by reference values) and maternal characteristics. Because of the lack of established and validated cutoff values, we had to use reference ranges determined from presumably healthy adult individuals to define fatty acid categories. These ones could be different in a pregnant population and are limited by the fact that they do not have an underlying relation to health status. Reference values have still the advantage to be specific of laboratory protocol for the analysis of erythrocyte fatty acids.

## Conclusions

In conclusion, this study revealed a striking low monounsaturated and long-chain omega-3 polyunsaturated fatty acid status in maternal erythrocytes at the first weeks of pregnancy. LCPUFA deficiencies at an early time are critical not only because it is a crucial step for fetal development but also because LCPUFA levels will continue to decline throughout pregnancy. Cutoff values are warranted to identify, as soon as possible, pregnant women who are at increased risk of adverse maternal and/or fetal health outcomes as a result of either under- or overexposure to fatty acids and those who are not at risk of these outcomes. This establishment of such values will not be easy taking into account the variety of methods of collection, biological samples and analytical techniques used to measure fatty acid status. Even if studies which investigated implications of fatty acid status in maternal, fetal and infant health have multiplied during the last few decades, there is still no consensus regarding specific recommendations to provide to patients. Because metabolism of PUFA is competitive, the evaluation of n-6 and n-3 PUFA should be considered simultaneously. Data suggest also that tFA may be a confounding variable in such studies. The influence of fatty acid on pregnancy outcomes is probably not related to a single unique fatty acid: it is the global profile that is at stake. High prevalence of inadequate status for several fatty acids in pregnant women, as observed in this study, could become a public health issue, requiring public health interventions. Finally, based on the demonstrated health impact of fatty acids, particularly omega-3 LCPUFA, delivered from the mother to the foetus, we strongly believe that both clinicians and patients should be aware of the importance of an optimal fatty acid status at the beginning of pregnancy.

## Additional file

Additional file 1: **Table S1.** Pearson correlation coefficients between polyunsaturated fatty acids as well as between *trans* fatty acids and polyunsaturated fatty acids. (DOC 40 kb)

## Abbreviations

AA: Arachidonic acid
ALA: Alpha-linolenic acid
AO: Oleic acid
DGLA: Dihomo-gamma-linolenic acid
DHA: Docosahexaenoic acid
El: Elaidic acid
EPA: Eicosapentaenoic acid
FA: Fatty acids
FAME: Fatty acid methyl esters
FFQ: Food frequency questionnaire
GLA: Gamma-linolenic acid
IOM3: Omega-3 index
LA: Linoleic acid
LCPUFA: Long-chain polyunsaturated fatty acids
MUFA: Monounsaturated fatty acids
Myr: Myristic acid
n-3 PUFA: Omega-3 polyunsaturated fatty acids
n-6 PUFA: Omega-6 polyunsaturated fatty acids
Pal: Palmitic acid
Pol: Palmitoleic acid
PPARs: Peroxisome proliferator-activated receptors
PUFA: Polyunsaturated fatty acids
RV: Reference values
SFA: Saturated fatty acids
Ste: Stearic acid
tFA: *Trans* fatty acids
Tvac: *Trans* vaccenic acid
Vac: *Cis* vaccenic acid
