# Supplementary material for: Low omega-3 index values and monounsaturated fatty acid levels in early pregnancy: an analysis of maternal erythrocytes fatty acids
Source: Lipids Health Dis. 2018 Apr 2;17:63. doi: 10.1186/s12944-018-0716-6 (PMC5879610; doi:10.1186/s12944-018-0716-6)
Supplement: Supplementary file 1 — Table S1. Pearson correlation coefficients between polyunsaturated fatty acids as well as between trans fatty acids and polyunsaturated fatty acids. (DOC 40 kb) [file 12944_2018_716_MOESM1_ESM.doc]

**Supplementary Material.** Pearson correlation coefficients between polyunsaturated fatty acids as well as between *trans* fatty acids and polyunsaturated fatty acids

| **Variables** | **n-6 PUFA** | | | |  | **n-3 PUFA** | | |  | **tFA** | |  | **IOM3** |
| --- | --- | --- | --- | --- | --- | --- | --- | --- | --- | --- | --- | --- | --- |
|  | **LA** | **GLA** | **DGLA** | **AA** |  | **ALA** | **EPA** | **DHA** |  | **Tvac** | **El** |  |  |
| LA | - |  |  |  |  |  |  |  |  |  |  |  |  |
| GLA | **0.18** | - |  |  |  |  |  |  |  |  |  |  |  |
| DGLA | 0.10 | **0.18** | - |  |  |  |  |  |  |  |  |  |  |
| AA | **-0.24** | -0.08 | -0.13 | - |  |  |  |  |  |  |  |  |  |
| ALA | 0.10 | -0.11 | 0.07 | 0.09 |  | - |  |  |  |  |  |  |  |
| EPA | -0.12 | -0.07 | **-0.25** | **-0.51** |  | 0.01 | - |  |  |  |  |  |  |
| DHA | **-0.27** | -0.06 | **-0.27** | **-0.48** |  | **-0.28** | **0.70** | - |  |  |  |  |  |
| Tvac | 0.01 | 0.04 | -0.09 | -0.03 |  | -0.04 | 0.10 | 0.15 |  | - |  |  |  |
| El | -0.01 | -0.03 | -0.02 | 0.02 |  | 0.11 | -0.03 | -0.12 |  | **0.54** | - |  |  |
| IOM3 | **-0.26** | -0.06 | **-0.28** | **-0.50** |  | **-0.24** | **0.77** | **0.99** |  | 0.15 | -0.11 |  | - |

Data are presented as Pearson correlation coefficient.

Bold font indicates P values less than 0.05.
